# Novel inflammatory and insulin resistance indices provide a clue in cerebral amyloid angiopathy

**DOI:** 10.1038/s41598-024-62280-z

**Published:** 2024-05-20

**Authors:** Hang-hang Zhu, Yun-chao Wang, Liu-chang He, Hai-yang Luo, Ce Zong, Ying-hao Yang, Jing-Hao Wu, Bo Song, Yuan Gao, Yu-ming Xu, Yu-sheng Li

**Affiliations:** 1 Department of Neurology, The First Affiliated Hospital of Zhengzhou University, Zhengzhou, Henan China; 2 NHC Key Laboratory of Prevention and Treatment of Cerebrovascular Diseases, Zhengzhou, China

**Keywords:** Cerebral small vessel disease, Cerebral amyloid angiopathy, Hypertensive arteriopathy, Inflammation, Neutrophil–lymphocyte ratio, Triglyceride–glucose index, Biomarkers, Diseases, Neurology, Risk factors

## Abstract

This study investigated the correlation of newly identified inflammatory and insulin resistance indices with cerebral amyloid angiopathy (CAA), and explored their potential to differentiate CAA from hypertensive arteriopathy (HA). We retrospectively analyzed 514 consecutive patients with cerebral small vessel disease (CSVD)-related haemorrhage, comparing the differences in novel inflammatory and insulin resistance indices between patients with CAA and HA. Univariate regression, LASSO and multivariate regression were used to screen variables and construct a classification diagnosis nomogram. Additionally, these biomarkers were explored in patients with mixed haemorrhagic CSVD. Inflammatory indices were higher in CAA patients, whereas insulin resistance indices were higher in HA patients. Further analysis identified neutrophil-to-lymphocyte ratio (NLR, OR 1.17, 95% CI 1.07–1.30, *P* < 0.001), and triglyceride–glucose index (TyG, OR = 0.56, 95% CI 0.36–0.83, *P* = 0.005) as independent factors for CAA. Therefore, we constructed a CAA prediction nomogram without haemorrhagic imaging markers. The nomogram yielded an area under the curve (AUC) of 0.811 (95% CI 0.764–0.865) in the training set and 0.830 (95% CI 0.718–0.887) in the test set, indicating an ability to identify high-risk CAA patients. These results show that CSVD patients can be phenotyped using novel inflammatory and insulin resistance indices, potentially allowing identification of high-risk CAA patients without haemorrhagic imaging markers.

## Introduction

Sporadic cerebral small vessel disease (CSVD), a prevalent age-associated pathological condition, is responsible for ischaemic stroke and intracerebral haemorrhage, representing a leading cause of vascular cognitive impairments and dementia^1,2^. CSVD has two primary aetiological subtypes: cerebral amyloid angiopathy (CAA) and hypertensive arteriopathy (HA)^3^. CAA is characterized by the deposition of amyloid-β (Aβ) within small cortical and leptomeningeal vessels^4,5^. Conversely, HA is predominantly characterised by arteriolosclerosis due to aging, hypertension, and other conventional vascular risk factors^5^. Accurate differentiation between these two conditions is vital for clinical decision-making, as they exhibit marked differences in clinical implications and prognoses^6^.

Performing standardised histopathological examinations is often challenging; however, magnetic resonance imaging (MRI) has potential to distinguish CAA from HA as both have distinct characteristic haemorrhagic neuroimaging markers^7^. Multiple lobar haemorrhages (intracerebral haemorrhage and/or cerebral microbleeds) and/or superficial cortical siderosis (cSS) are indicative of CAA^8^. In contrast, haemorrhages located in deeper brain structures, such as the basal ganglia, thalamus, or brain stem, are indicative of HA^9^.

However, the dependence of MRI on haemorrhagic imaging markers restricts its clinical application. In fact, complete MRI sequences are not available for all patients with CSVD, especially susceptibility-weighted imaging (SWI), which is very sensitive in detecting cerebral microbleeds^10,11^. In addition, nearly 40% of patients with CSVD display mixed lobar and deep hemorrhages^12–14^, leaving them unclassifiable as CAA or HA based on current clinical-radiological criteria^15^. Notably, following the progress of clinical and multiomics studies, the performance of non-imaging markers in CSVD has attracted considerable attention. Inflammation and insulin resistance (IR) have been shown to be involved in the occurrence and progress of CSVD, and even show different manifestations in different subtypes^16–19^. Meanwhile, new indices such as neutrophil to lymphocyte ratio (NLR), platelet to lymphocyte ratio (PLR), lymphocyte to monocyte ratio (LMR) and systemic immunity-inflammation index (SII) can suitably reflect the inflammation status^20–22^, while triglyceride glucose (TyG) index is suggested as a convenient marker for IR since it proved to be a simple measure of insulin sensitivity^23,24^.

In this study, we explored and compared the variations in multiple biomarkers, especially novel inflammatory and IR indices, between the CAA and HA groups to identify potential indicators that could indicate their pathological backdrop and construct a nomogram. Subsequently, under the assumption that the presence of mixed haemorrhages does not rule out a CAA diagnosis, we further examined the performance of these biomarkers in patients with CSVD and mixed haemorrhages to ascertain whether they could assist in producing a more precise and pathologically pertinent classification of these patients.

In summary, our study evaluated the effectiveness of these novel inflammatory and IR indices for differentiating CSVD subtypes, with the goal of establishing a straightforward, reliable, and quantitative diagnostic model to determine the underlying microvascular types of CSVD.

## Methods

### Subjects

Consecutive patients were enrolled from the First Affiliated Hospital of Zhengzhou University CSVD registry between January 2016 and March 2023 (n = 1839). To minimize patient heterogeneity and enable comparison, we examined the brain MRI findings of all CSVD patients and recruited those who met the following study criteria (1)presence of two or more CSVD-related imaging manifestations, which were evaluated according to the Standards for Reporting Vascular Changes on Neuroimaging (STRIVE)^25^; (2) complete MRI scan images, especially SWI and/or T2*-weighted gradient echo (GRE) imaging; (3) presentation with MRI-visible haemorrhagic lesions, including cerebral microbleeds (CMBs) and intracerebral haemorrhage (ICH); (4) absence of other cause of haemorrhagic lesions, such as head trauma, arteriovenous malformation, haemorrhagic tumour, CNS vasculitis, etc.

The final cohort comprised 514 participants. Based on the locations of haemorrhagic lesions, patients with strictly lobar haemorrhages involving the cerebral cortex and underlying white matter were coded as having CAA per Boston criteria version 2^8^; patients with strictly deep haemorrhages in the basal ganglia, thalamus, or brainstem were diagnosed with HA (non-CAA); and patients with both lobar and deep CMBs with or without ICHs were diagnosed with a Mix. Cerebellar haemorrhagic lesions were not included in this classification because of their ambiguous nature^26^.

### MRI assessment and SVD scores

Two experienced neurologists independently evaluated both the non-haemorrhagic and haemorrhagic markers of CSVD, aligning their assessments with the Standards for Reporting Vascular Changes in Neuroimaging consensus criteria^27^. We employed two validated composite scoring systems predicted on four recognised MRI markers of SVD to collect and semi-quantify the global CAA- and HA-specific pathological burdens, denoted as the CAA-SVD and HA-SVD scores, respectively^28,29^. Briefly, in the CAA-SVD scoring (ordinal scale 0–6), one point was assigned each for 2–4 lobar CMBs, ≥ 20 centrum semiovale perivascular spaces (CSO-EPVSs), presence of ≥ 2 deep white matter hyperintensities (WMH) or 3 periventricular WMH or focal cortical superficial siderosis (CSSs), whereas two points were assigned each for ≥ 5 lobar CMBs or disseminated CSSs^30,31^. In HA-SVD scoring (ordinal scale 0–4), one point each was attributed for ≥ 1 deep CMBs, ≥ 1 LI, ≥ 10 basal ganglia perivascular spaces (BG-EPVSs), and the presence of ≥ 2 deep WMH or 3 periventricular WMH^32,33^.

### Clinical assessment

Upon enrolment in the study, we gathered the following data from the patients: demographics, risk factors (blood pressure, alcohol consumption, and smoking status), medical history (hypertension, diabetes, dyslipidemia, peripheral or cardiac vasculopathy, and atrial fibrillation), and information regarding medication use (antiplatelet and anticoagulant). Within 48 h of the patient's MRI examination, baseline fasting venous blood samples were obtained for various laboratory tests, including routine blood examination and measurements of total cholesterol (TC), triglyceride (TG), high-density lipoprotein cholesterol (HDL-C), low-density lipoprotein cholesterol (LDL-C), fasting blood glucose (FBG), and homocysteine levels. Blood tests were performed near the time of acute ICH. To exclude the effect of acute cerebral ischemia or hemorrhage on blood examination indicators, we verified that the enrolled patients had no new cerebrovascular events within 48 h before and after the blood test.

We calculated the NLR, the PLR, and the LMR using from the absolute ratios of the corresponding blood cell counts. The systemic immune inflammation index (SII) was determined as the product of the platelet and neutrophil counts divided by the lymphocyte count. The monocyte/HDL-C ratio (MHR), neutrophil/HDL-C (NHR), and TG/HDL-C ratios were derived by dividing the absolute monocyte count, neutrophil count, or TG by the HDL-C value^20,21^. Finally, the TyG index was computed on a logarithmic scale of [TG (mg/dL) × fasting glucose (mg/dL)/2]^34–36^.

### Statistical analysis

The normality of continuous variables was assessed using a QQ-plot. Continuous variables that adhered to a normal distribution were reported as means and standard deviations and analyzed using a t-test for two-group comparisons. Conversely, non-normal continuous variables are represented as medians and interquartile ranges (IQR), and the Wilcoxon rank-sum test and Kruskal–Wallis test were used when comparing 2 or 3 groups, respectively. Pearson’s chi-square test was used to analyze categorical data.

Variables that were statistically and clinically significant in the univariate analysis were subjected to least absolute shrinkage and selection operator (LASSO) regression analysis. This was performed to identify risk factors more effectively and manage the linear effects. The CAA and HA groups were randomly allocated to the training and test sets in a 7:3 ratio using a computer-generated fixed ratio. The training and test sets were used for model construction and validation, respectively. LASSO regression compresses the coefficient estimates towards zero, and the extent of compression is dependent on an additional parameter, λ. As λ increases, the model's compression intensifies, the number of independent variables entering the model reduces, and the model's capability to select the main variable improves. The optimal value of λ was determined through tenfold cross-validation. The selected variables were then analyzed using multivariate logistic regression to determine the independent risk factors, and false discovery rate analysis was performed. The results are reported as odds ratios (ORs) and 95% confidence intervals (CIs), and the AUC of the ROC analysis was used to assess the performance of the model. Subsequently, a diagnostic nomogram was constructed based on independent prognostic factors. This nomogram served to visualize the results and guide the classification of CSVD under ideal conditions. A concordance index (C-index) was calculated to assess the discriminative power of the model. The nomogram was calibrated by using a calibration curve. Decision curve analysis (DCA) was also performed to verify the clinical usefulness of the developed nomogram by determining its net benefits under varying threshold probabilities^36,37^. All experiments/ methods were performed in accordance with the relevant guidelines and regulations^38^.

### Ethical statements

This study was approved by the Ethics Committee of the First Affiliated Hospital of Zhengzhou University, China (No. 2021KY-0067-001). Informed consent was obtained from all participants and/or their legal guardians (s) for study participation, and written informed consent was obtained from the patients for their anonymised information to be published in this article.

## Results

### Clinical, neuroimaging and laboratory characteristics of patients with CSVD

After applying the inclusion and exclusion criteria, the final cohort included 171 patients with probable CAA (33%), 207 patients with HA (40%), and 139 patients with mixed hemorrhagic lesions (27%). Initially, we focused on patients with relatively definite diagnoses. Tables 1 and 2 present the demographic, imaging, and laboratory characteristics of the patients with CAA and HA (non-CAA). The data for the mixed hemorrhagic lesion group are discussed in subsequent analyses owing to their complex lesion backgrounds and lower diagnostic certainty. In the demographic data, probable CAA patients were found to be older and had a higher likelihood of cognitive impairment, dementia, and transient focal neurological episodes (TFNEs). In contrast, the HA group had a higher prevalence of vascular risk factors, including hypertension, diabetes, and dyslipidemia, and a higher likelihood of anxiety or depression.

**Table 1:** Demographics and clinical characteristics of the probable cerebral amyloid angiopathy (CAA) and hypertensive arteriopathy (HA).

	CAA (n = 171)	HA (n = 207)	*P *value
Demographic characteristics			
Age, years	67 (60,74)	65 (58,70)	0.018*
Male, n (%)	93 (54.39%)	126 (61.17%)	0.184
Vascular risk factors, n (%)			
Hypertension	90 (52.63%)	163 (79.13%)	< 0.001*
Diabetes mellitus	33 (19.3%)	78 (37.86%)	< 0.001*
Dyslipidemia	17 (9.94%)	58 (28.16%)	< 0.001*
Peripheral or cardiac vasculopathy	26 (15.2%)	24 (11.65%)	0.171
Atrial fibrillation	15 (8.77%)	11 (5.34%)	0.190
Smoking	35 (20.47%)	60 (29.13%)	0.054
Drinking	27 (15.79%)	53 (25.73%)	0.019*
Anticoagulation or antiplatelet use history	39 (22.81%)	45 (21.84%)	0.823
Major clinical events, n (%)			
Cognitive impairment or dementia	36 (21.1.%)	16 (7.8%)	< 0.001*
Anxiety or depression	9 (5.3%)	30 (14.6%)	0.003
TFNE	14 (8.2%)	0 (0.0%)	< 0.001*
Neuroimaging characteristics			
-Hemorrhagic			
≥ 5 CMB, n (%)	89 (52.4%)	30 (14.6%)	< 0.001*
ICH (non-acute), n (%)	80 (47.3%)	8 (3.9%)	< 0.001*
cSS presence, n (%)	57 (33%)	0 (0%)	< 0.001*
Cerebellum CMB presence, n (%)	41 (24.3%)	20 (9.7%)	< 0.001*
-Non-hemorrhagic			
≥ 20 CSO-EPVS, n (%)	58 (33.9%)	29 (14.1%)	< 0.001*
≥ 20 BG-EPVS, n (%)	64 (37.4%)	97 (47.1%)	0.059
Moderate to severe WMH, n (%)	80 (46.8%)	75 (36.4%)	0.042*
Multiple lacunes, n (%)	111 (64.9%)	174 (84.5%)	< 0.001*
Clinical assessment			
Systolic pressure, mmHg	136.98 ± 20.36	141.08 ± 16.14	0.014*
Diastolic pressure, mmHg	81.52 ± 12.73	82.23 ± 10.50	0.134
Heart rate	78 (72,82)	78 (75,80)	0.901

**Table 2:** Laboratory data and derived inflammatory and insulin resistance indices of the probable cerebral amyloid angiopathy (CAA) and hypertensive arteriopathy (HA).

	CAA (n = 171)	HA (n = 207)	*P *value
Laboratory data			
White blood cell count, 10^9^/L	6.69 (5.34, 7.98)	6.2 (5.2, 7.57)	0.111
Platelet count, 10^9^/L	205.5(162.75, 241.75)	211.0 (172.0, 240.0)	0.377
Neutrophil count, 10^9^/L	4.08 (3.27, 5.77)	3.83 (3.02, 4.97)	0.021*
Lymphocyte count, 10^9^/L	1.50 (1.05, 1.98)	1.72 (1.35, 2.04)	0.001*
Monocyte count, 10^9^/L	0.45 (0.34, 0.55)	0.44 (0.34, 0.53)	0.639
Eosinophil count, 10^9^/L	0.10 (0.04, 0.187)	0.10 (0.06, 0.180)	0.124
FBG, mmol/L	5.03 (4.46, 5.85)	5.23 (4.78, 6.33)	0.005*
TC, mmol/L	3.92 (3.09, 4.68)	3.91 (3.23, 4.69)	0.842
TG, mmol/L	1.08 (0.79, 1.53)	1.26 (0.96, 1.62)	0.005*
HDL-c, mmol/L	1.13 (0.9, 1.36)	1.12 (0.92, 1.33)	0.73
LDL-c, mmol/L	2.26 (1.68, 3.03)	2.26 (1.68, 2.9)	0.944
Uric Acid, μmol/L	248.5 (195, 305.5)	277.5 (226, 338)	0.001*
Creatinine, μmol/L	65 (52, 77)	67 (58, 78)	0.062
Urea, mmol/L	5.1 (4.25, 6.07)	5.1 (4.2, 6.1)	0.962
AST, U/L	21 (16, 26)	20 (17, 25)	0.338
ALT, U/L	17 (12, 26)	19 (14, 26)	0.113
Homocysteine, μmol/L	14.21 (11.13, 18.70)	13.7 (11.5, 16.63)	0.377
Inflammatory and insulin resistance indices			
NLR	2.85 (2.00, 4.36)	2.25 (1.73, 3.09)	< 0.001*
PLR	135.99 (101.7, 180.76)	124.93 (99.59, 155.58)	0.027*
MLR	0.28 (0.22, 0.42)	0.26 (0.20, 0.34)	0.005*
SII	579.04 (362.06, 890.9)	461.54 (334.31, 696.92)	0.010*
MHR	0.4 (0.3, 0.52)	0.39 (0.28, 0.52)	0.733
NHR	3.93 (2.58, 5.16)	3.51 (2.35, 5.16)	0.201
TyG	8.47 (8.07, 8.83)	8.57 (8.29, 8.97)	0.001*
TG/HDL-C	0.90 (0.63, 1.46)	1.12 (0.79, 1.60)	0.014*

Regarding the imaging characteristics, our findings are consistent with those of previous studies. CAA patients were more likely to exhibit moderate to severe (≥ 20) MRI-visible CSO-PVS^39^, moderate to severe WMH^40^, and multiple (≥ 5) CMBs. Further, cSS and convexity subarachnoid haemorrhage (cSAH) were observed only in the CAA group^41^. In contrast, patients with HA showed a higher likelihood of having multiple lacunar infarcts (LI), which is a typical feature of spontaneous HA.

In the laboratory tests, probable CAA patients had a significantly higher neutrophil count, while patients with probable HA had a higher had a higher lymphocyte count. Additionally, patients with probable HA had higher systolic blood pressure and FBG, TG, and uric acid levels, mirroring their higher prevalence of hypertension, diabetes, and lipid metabolism disorders. Furthermore, we evaluated the novel composite indices of inflammation and glucolipid metabolism to gain insights into the inflammatory and IR profiles of the two groups. Probable CAA patients had significantly higher inflammatory composite indices, including NLR, PLR, LMR, and SII, than probable HA patients. In contrast, the insulin resistance indices, TyG and TG/HDL-C^42^, were significantly higher in patients with HA than in those with CAA.

### Variables screening based on univariate analysis and LASSO regression

**Figure 1:**
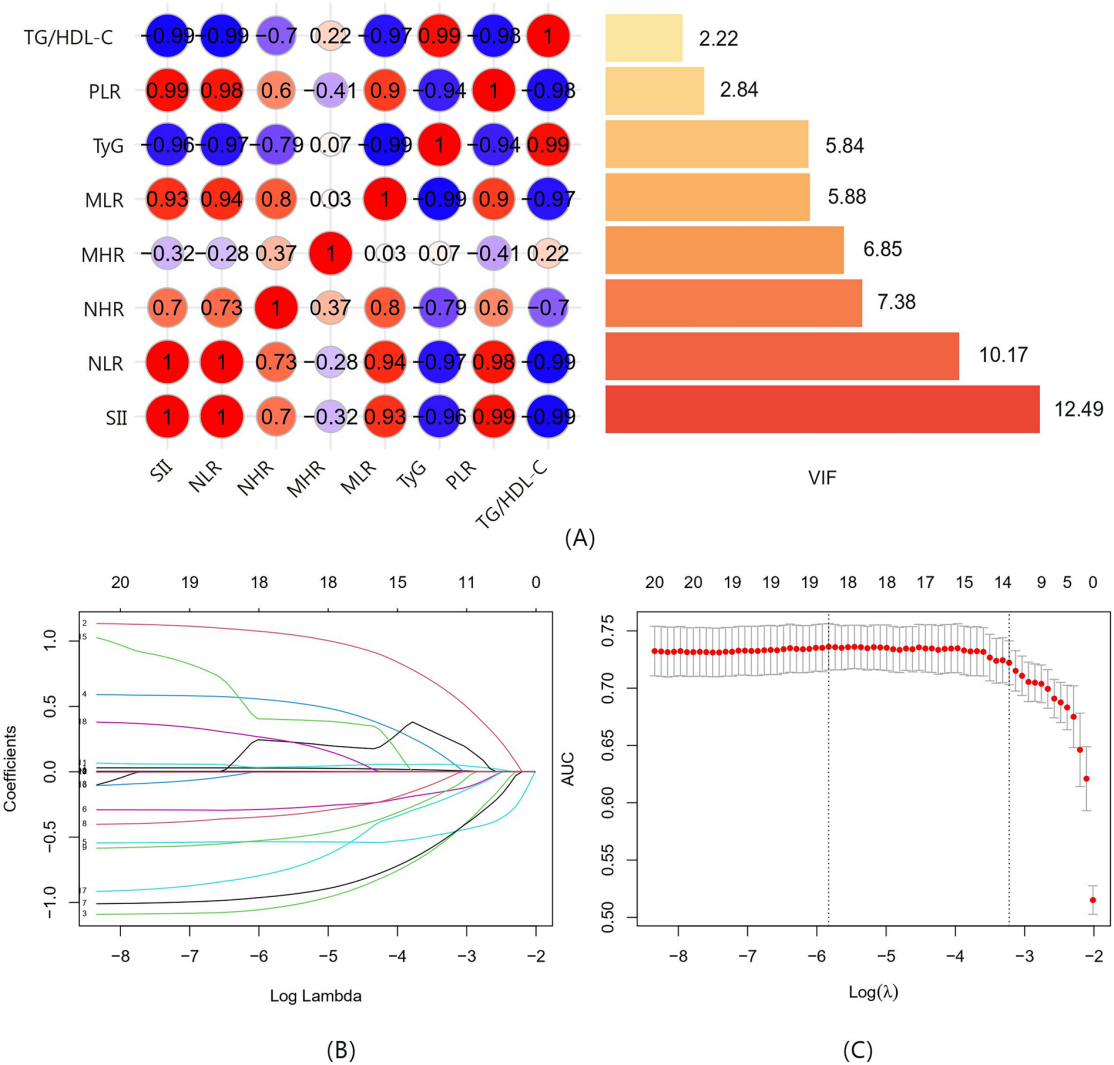
(**A**) Correlation and collinearity analysis between markers of inflammation and insulin resistance between CAA and HA. The left side shows the correlation heatmap of each index, and the right side shows the VIF values obtained from collinearity analysis. (**B**,**C**) The Least absolute shrinkage and selection operator (LASSO) binary logistic regression model for identifying independent risk factors. (**B**) LASSO coefficient profiles of differential characteristics screened by univariate analysis. Each colored line represents the coefficient of each characteristic. (**C**) Plots of the results of cross-validation: the red dots in the figure represent the target parameters corresponding to each lambda.

The results of the multicollinearity analysis are presented in Fig. 1A. To eliminate interference from multicollinearity and simplify the model, univariate analysis combined with LASSO regression was used to screen and identify potential features. Figure 1B illustrates the variation in regression coefficients of each variable with different λ, and λ-min (λ-min = 0.03626291) was chosen as the optimal value (Fig. 1C). Nine variables with non-zero coefficients were selected, including age, hypertension, dyslipidemia, drinking, ≥ 20 CSO-EPVS, moderate to severe WMH, NLR, multiple LI, and TyG.

### Multivariate logistic regression and nomogram development

Table 3 shows the results of the multivariate logistic regression model for CSVD classification. After adjusting for potential confounders, age, ≥ 20 CSO-PVS, moderate to severe WMH, and NLR were identified as independent risk factors for CAA. In contrast, hypertension, dyslipidemia, drinking, multiple LI, and TyG are indicative of HA.

**Table 3:** Factors independently associated with CAA.

Variables	OR	95% CI		*P* value	FDR
Age	1.038	1.01	1.067	0.007**	0.013
Hypertension	0.493	0.289	0.84	0.009**	0.012
Dyslipidemia	0.387	0.2	0.749	0.005**	0.015
Drinking	0.413	0.215	0.794	0.008**	0.012
Multiple LI	0.277	0.149	0.515	< 0.001***	0.003
Moderate-to-severe WMH	1.934	1.152	3.248	0.013*	0.015
≥ 20 CSO-EPVS score	3.439	1.916	6.171	< 0.001***	< 0.001
NLR	1.155	1.043	1.279	0.006**	0.014
TyG	0.604	0.397	0.919	0.019*	0.019

*Represents *P* < 0.05, **Represents *P* < 0.01, ***Represents *P* < 0.001.

**Figure 2:**
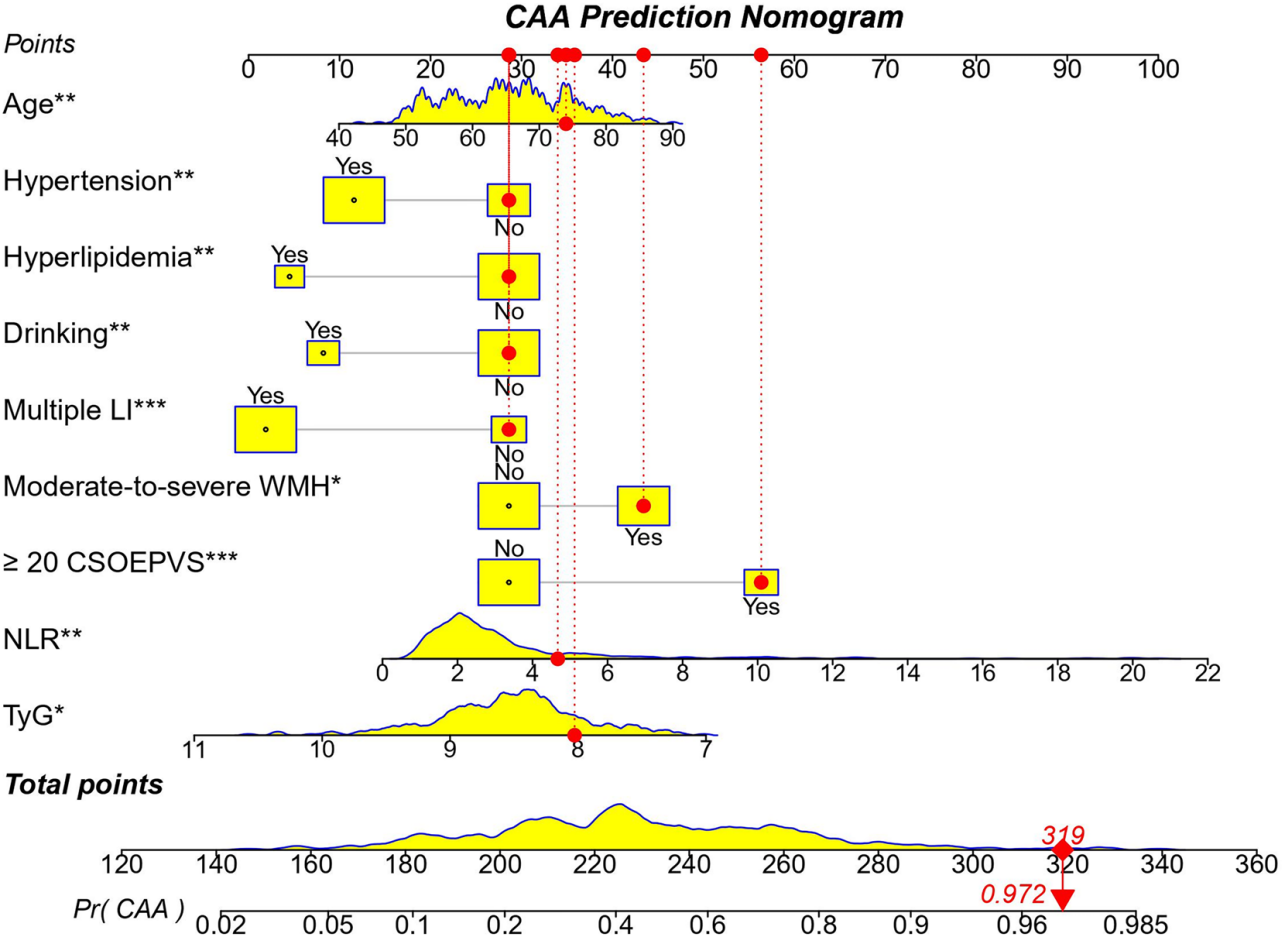
Overview of the nomogram to predict the probability of the risk of CAA in CSVD. The distribution of categorical variables is displayed by yellow boxes, and distribution of continuous variables is displayed by yellow density plots, the red dots and arrows represents an exemplar patient is shown as an example. (Observation = 112, CAA).

A nomogram based on non-hemorrhagic markers was constructed to visually represent the regression analysis results and facilitate rapid clinical judgment. Clinicians are able use this semiquantitative method to estimate the likelihood of a patient with CSVD being categorized as having CAA. Each parameter corresponded to a score, and a higher total score indicated a higher probability that the patient’s primary pathological classification was CAA. Conversely, a lower total score suggested an HA classification. An example of a training set (observed value = 112) is shown (Fig. 2). The nomogram score indicated a probability of CAA greater than 95%, and corresponding to this high probability was that the patient belonged to the CAA group.

**Figure 3:**
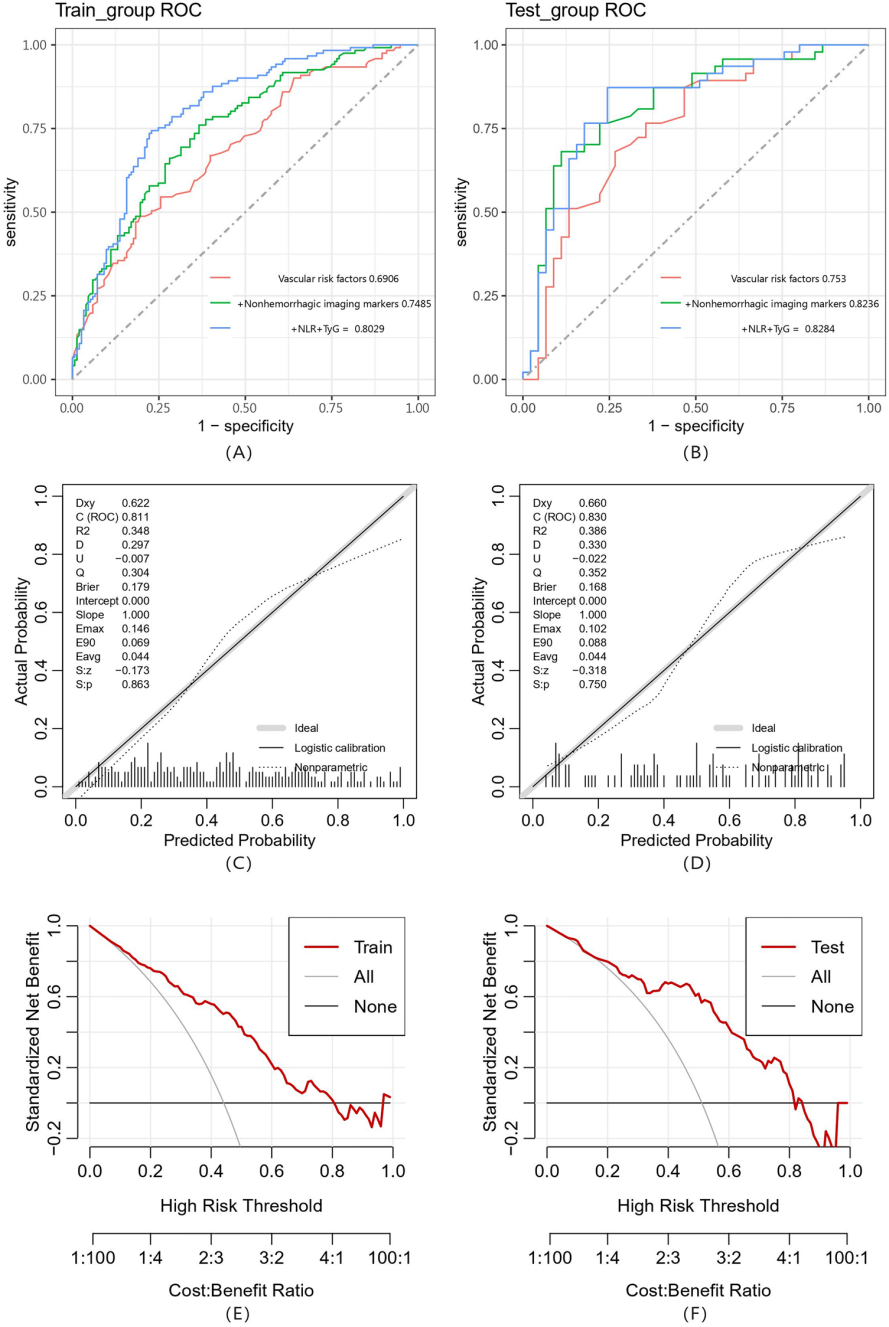
Evaluation and validation of the nomograms in the training and testing sets (**A**). (**B**) The area under the curve. (**C**,**D**) Internal consistency calibration plot. The abscissa is the Predicted Probablity, and the ordinate is the observed Actual Probablity. The diagonal line is the reference line, which is the case where the predicted value = the actual value. (**E**,**F**) Decision curve analysis (DCA). The y axis represents the net benefit and the x axis represents the high-risk thresholds that were chosen here range from 0 to 1. The black line represents the assumption of none have CAA, the gray line represents the assumption that all CSVD are diagnosed as CAA.

The discriminative power of the constructed nomogram was quantified using the receiver operating characteristic (ROC) curve and Harrell’s C-index. As shown in Fig. 3A,B, the area under the curve (AUC) for the training and test sets were 0.811 (90% CI 0.764–0.865) and 0.830 (90% CI 0.718–0.887), respectively, indicating good discriminatory power. A comparison of the discriminative abilities of various models revealed that the model that included the NLR and TyG exhibited the best performance in diagnosing CAA. The calibration curves for the nomogram, presented in Fig. 3C,D, run close to the diagonal for both the training and test sets, indicating a strong correlation between the predicted and observed values and hence a good calibration. Decision curve analysis (DCA) curves were further plotted to assess the clinical applicability of the nomogram^43^, as shown in Fig. 3E,F. The results showed that the net benefit of using the nomogram to predict the probability of CAA was greater when the threshold probability was < 0.80, suggesting its potential clinical use. However, we acknowledge that there is a major limitation: there are no PET scans, CSF studies, biopsy samples, or autopsy data to truly determine whether the nomogram performed well in differentiating CAA from HA.

### Performance of novel markers and nomograms in mixed hemorrhagic CSVD

Although previous studies have tended to classify CSVD patients with mixed haemorrhagic lesions as having fulminant or late-stage HA^44^, large bodies of data have shown that the pathological diagnosis of CAA cannot be excluded in some patients, and that CAA may even be the main pathological mechanism^45,46^. Therefore, we hypothesised that there may be some patients with CAA and some patients affected by the main pathological mechanisms of CAA and HA simultaneously in the mixed group, such that the mixed group may differ in some characteristics from the HA group but resemble the CAA group. We separately compared the demographic, imaging, and laboratory data of patients with CSVD and mixed haemorrhagic lesions with those of patients with either type of isolated bleeding (Supplementary Table 1).

**Figure 4:**
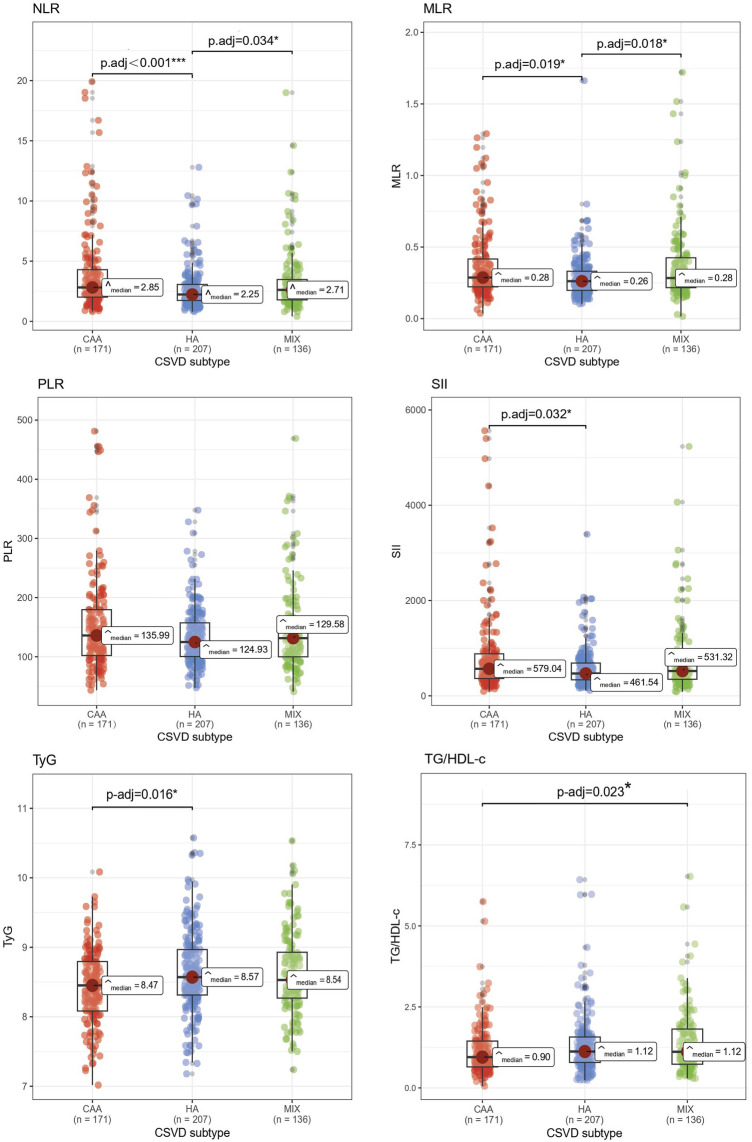
Performance of inflammatory and insulin resistance indices in cerebral amyloid angiopathy (CAA), hypertensive arteriopathy (HA) and mixed hemorrhagic CSVD(MIX). Significant adjusted *P* values (*P*-adj < 0.05) are indicated in the figure.

We focused on the markers of inflammation and IR, which were identified as potentially suggestive of the underlying pathological mechanisms. As demonstrated in Fig. 4, the Kruskal–Wallis test showed that, except for PLR, the other inflammatory and IR indices were significantly different among the three groups. Subsequently, we performed a pairwise comparison, most notably finding that the NLR and MLR levels of mixed hemorrhagic CSVD resembled those of CAA, both of which were significantly higher than those of HA.

Additionally, we evaluated the nomogram's performance in patients with CSVD and mixed hemorrhagic lesions, aiming to identify those with a high CAA risk. As stated earlier, each measure was assigned a score, which was then added and converted to the probability of CAA. We subsequently divided these patients into a high CAA-risk group (34, 25%) and a non-high CAA-risk group (102, 75%) using a 50% threshold. For each CSVD patient with mixed hemorrhagic lesions, we characterized the CAA-specific and HA-specific pathological burdens using the CAA-SVD and HA-SVD scores, respectively. Interestingly, we found that the CAA-SVD score of the high-CAA-risk group was significantly higher than that of the non-high-CAA-risk group (3[2, 4] vs. 2[2, 3], *P* < 0.001), whereas there was no significant difference in HA-SVD scores between the two groups (4[3, 4] vs. 3[3, 4], *P* = 0.273). In addition, the incidence of cSS in the CAA risk group was higher than that in the non-high-CAA-risk group (23.5% vs. 9.8%, *P* = 0.041). This indicates that this nomogram holds promise in detecting CAA-prone patients among patients with CSVD without interference from complex hemorrhagic manifestations.

## Discussion

In this cross-sectional study, we aimed to explore non-haemorrhagic imaging and molecular biomarkers for the clinical diagnosis of CAA and HA (non-CAA), focusing on novel inflammatory and IR composite indices. Our aim was to provide insights into the classification of patients with CSVD along the disease spectrum, thereby overcoming the limitations of hemorrhagic imaging markers.

Through a retrospective comparative analysis of clinical data from 171 probable CAA and 207 probable HA patients, we observed distinct tendencies in the category and distribution of non-haemorrhagic imaging burden between the two subtypes, consistent with previous reports^27^. Patients with CAA tended to have more severe CSO-EPVSs and WMH, whereas those with HA were more strongly associated with multiple lacunes. In terms of laboratory data, high NLR, PLR, LMR, and SII may be associated with CAA, whereas high TyG and TG/HDL-C index may indicate HA. The variability in the association between these composite indices and the different forms of CSVD suggests that inflammatory and IR abnormalities exert different effects and lead to different vascular injury outcomes in the CSVD spectrum^47^.

Specifically, systemic inflammation, represented by NLR and other inflammatory composite indices, is preferentially associated with CAA-related vascular injury. Recent studies using mouse models demonstrated a bidirectional association^48^. This may be because the accumulation of β-amyloid plaques in the cerebral cortex triggers an immune response, which ultimately results in an inflammatory response^49^. It is worth mentioning that inflammation also has been reported in HA but seems to be seems to favor vascular inflammatory responses/endothelial dysfunction^43^, which may be linked to underlying hypertensive arteriopathology and commonly reflected by homocysteine, vascular endothelial growth factor, E-selectin, P-selectin, etc^50,51^. Regional analysis have also previously revealed that blood markers of vascular inflammation were frequently associated with HA, whereas blood markers of systemic inflammation we focused on here appeared to correlate with CAA^47,52^. In contrast, the TyG index, a simple and reliable surrogate indicator of insulin resistance, indicates endothelial dysfunction, reduced nitric oxide (NO) bioavailability, and increased endothelin-1 (ET-1) secretion. These alterations could lead to functional inhibition or even disruption of the blood–brain barrier, which is consistent with the mechanism of HA-related pathological damage. LASSO regression and multivariate logistic regression analysis showed that ≥ 20 CSO-EPVS, moderate to severe WMH, and high NLR were all independent risk factors for CAA, while hypertension, dyslipidemia, drinking, multiple LI, and TyG were more likely to be independent risk factors for HA. These indicators were used to construct a nomogram model to evaluate the propensity of CSVD patients to develop CAA. The patients were randomly divided into training and test sets, and Harrell’s C-index, calibration curve, ROC curve, and DCA were used to evaluate the model's identification, calibration, prediction ability, and clinical effectiveness in the two sets, respectively. Ultimately, the model demonstrated good discriminatory ability and the potential for clinical application.

Furthermore, we conducted a relevant comparative study on a specific yet uncommon group of patients with CSVD and mixed lesions. Collectively, we confirmed that the mixed hemorrhage group demonstrated greater similarities with patients with HA in terms of vascular risk profiles. These results are in line with those of some recent studies suggesting that CSVD with mixed hemorrhage presents a clinical phenotype sharing more similarities with probable HA patients than with CAA patients. Interestingly, their inflammatory composite indices were similar to those of CAA patients, and significantly higher than those of HA patients^14^. When mixed hemorrhage patients were scored with the nomogram, although most patients (75%) were more likely to be classified as HA based on their scores, a minority (25%) had total scores suggesting that they were more likely to have a pathological background similar to that of CAA. These patients were validated as having a significantly higher CAA-SVD burden, which was consistent with the nomogram results. This supports our hypothesis that mixed hemorrhage should not be assumed to be a manifestation of advanced HA. However, some patients may have varying degrees of CAA-related lesions. Notably, the nomogram we constructed has promise for identifying patients at high risk of CAA from CSVD patients with mixed hemorrhage, overcoming disturbances and limitations of imaging.

The strengths of this study include the enrolment of a relatively large number of CSVD patients with hemorrhagic lesions, which increased the robustness of the results, as well as the use of straightforward routine MRI base sequences and blood biochemical tests, which ensure the results are easily translatable into clinical practice. The proposed nomogram is simple and quick to apply, potentially assisting in identifying the major microvascular background of patients with CSVD and screening for high-risk CAA patients, thus serving as a valuable complement to the Boston criteria. However, our study has certain limitations. First, because this was a retrospective analysis, the MRI scanning protocols were not completely harmonized, and different imaging parameters and sequences may have affected the evaluation of CSVD markers. Moreover, other potential markers were not adequately collected and analyzed because some laboratory markers were not tested in many patients. Second, our study was limited to the Han Chinese population, necessitating further external validation in a larger cohort of patients is required. In addition, because our study was retrospective, longitudinal studies are required to validate our conclusions. Further investigation may reveal stronger associations between these inflammatory factors, IR, and different forms of CSVD.

## Conclusion

Overall, the results of this study suggest that full consideration of inflammatory and IR indices in clinical research and CSVD patient care may help to elucidate the complex vascular pathogenic mechanisms in the CSVD spectrum. We provide a new idea for screening high-risk patients with CAA without relying on haemorrhagic imaging markers, which may guide clinicians in further etiologic investigations and/or repeated neuroimaging to achieve precise intervention and treatment.

### Supplementary Information

Supplementary Table 1.

## Data Availability

The datasets generated and/or analysed in the current study are available from the corresponding authors (Yu-ming Xu, xuyuming@zzu.edu.cn and Yu-sheng Li, fccliyusheng@zzu.edu.cn) upon reasonable request.
