# Supplementary material for: Novel inflammatory and insulin resistance indices provide a clue in cerebral amyloid angiopathy
Source: Sci Rep. 2024 May 20;14:11474. doi: 10.1038/s41598-024-62280-z (PMC11106308; doi:10.1038/s41598-024-62280-z)
Supplement: Supplementary file 1 — Supplementary Table 1. [file 41598_2024_62280_MOESM1_ESM.docx]

[Supplementary](javascript:;) table 1: Demographics and clinical characteristics of cerebral amyloid angiopathy (CAA), hypertensive arteriopathy (HA) and mixed hemorrhagic cerebral small vessel disease (MIX).

|  | Mix(n=136) | Mix-CAA(n=171)  P. value | Mix-HA(n=207)  P. value |
| --- | --- | --- | --- |
| Demographic characteristics |  |  |  |
| Age, years | 64(57,73) | 0.069 | 0.788 |
| Male, n (%) | 96(70.6%) | 0.002 | 0.074 |
| Vascular risk factors, n (%) |  |  |  |
| Hypertension | 110(80.9%) | <0.001 | 0.692 |
| Diabetes mellitus | 43(31.6%) | 0.013 | 0.327 |
| Dyslipidemia | 24(17.6%) | 0.049 | 0.026 |
| Peripheral or cardiac  vasculopathy | 13(9.6%) | 0.081 | 0.542 |
| Atrial fibrillation | 9(6.6%) | 0.485 | 0.622 |
| Smoking | 34(25%) | 0.345 | 0.403 |
| Drinking | 31(22.8%) | 0.119 | 0.537 |
| Anticoagulation or  antiplatelet use history | 39(28.7%) | 0.241 | 0.151 |
| Major clinical events, n (%) |  |  |  |
| Cognitive impairment or dementia | 23(16.9%) | 0.360 | 0.009 |
| Anxiety or depression | 18(13.2%) | 0.014 | 0.729 |
| TFNE | 11(8.1%) | 0.975 | <0.001 |
| Clinical assessment |  |  |  |
| SBP, mmHg | 145(131,152) | 0.003 | 0.250 |
| DBP, mmHg | 80（74，90） | 0.005 | 0.103 |
| Heart rate | 78(72,78) | 0.545 | 0.582 |
| Neuroimaging characteristics |  |  |  |
| -Hemorrhagic |  |  |  |
| >5 Lobar CMB, n (%) | 81(59.6%) | 0.188 | - |
| >5 Deep CMB, n (%) | 94(69.1%) | - | <0.001 |
| cSS presence, n (%) | 18(13.2%) | <0.001 | - |
| -Non-hemorrhagic |  |  |  |
| >20 CSO-PVS, n (%) | 27(20%) | 0.007 | 0.158 |
| >20 BG-PVS, n (%) | 90(66.2%) | <0.001 | 0.001 |
| Moderate to severe WMH, n (%) | 96(70.6%) | <0.001 | <0.001 |
| Multiple lacunes, n (%) | 130(95.6%) | <0.001 | 0.001 |
| Laboratory data |  |  |  |
| White blood cell [count](javascript:;), 10^9^/L | 6.35(5.43,8.11) | 0.836 | 0.201 |
| Neutrophil [count](javascript:;), 10^9^/L | 4.02(3.17,5.33) | 0.550 | 0.119 |
| Lymphocyte [count](javascript:;), 10^9^/L | 1.58(1.19,1.92) | 0.247 | 0.028 |
| Monocyte [count](javascript:;), 10^9^/L | 0.45(0.36,0.60) | 0.270 | 0.103 |
| Eosinophil, 10^9^/L | 205(163.0,251.0) | 0.630 | 0.697 |
| FBG, mmol/L. | 5.32(4.60,6.77) | 0.037 | 0.665 |
| TC, mmol/L | 3.9(3.22,4.69) | 0.125 | 0.06 |
| TG, mmol/L | 1.19(0.89,1.72) | 0.024 | 0.841 |
| HDL, mmol/L | 1.08(0.87,1.28) | 0.068 | 0.099 |
| LDL, mmol/L | 2.11(1.59,2.78) | 0.045 | 0.051 |
| Uric Acid, μmol/L | 277(220,334) | 0.005 | 0.721 |
| Creatinine, μmol/L | 70(59.5,82.5) | 0.001 | 0.062 |
| Urea, mmol/L | 5.2(4.6,6.45) | 0.145 | 0.128 |
| AST, U/L | 19(15.5,23.5) | 0.104 | 0.370 |
| ALT, U/L | 17(12.0,24.5) | 0.868 | 0.079 |
| Homocysteine, μmol/L | 14.61(12.53,19.12) | 0.132 | 0.006 |
| Composite inflammatory ratios |  |  |  |
| NLR | 2.71(1.76,3.64) | 0.261 | 0.021 |
| PLR | 129.58(97.16,166.52) | 0.321 | 0.344 |
| MLR | 0.28(0.22,0.44) | 0.899 | 0.004 |
| SII | 531.32(347.88,747.60) | 0.509 | 0.091 |
| MHR | 0.42(0.32,0.63) | 0.109 | 0.05 |
| NHR | 3.61(2.66,5.31) | 0.472 | 0.071 |
| TyG | 8.54(8.27,8.96) | 0.017 | 0.864 |
| TG/HDL-C | 1.12(0.71,1.84) | 0.013^*^ | 0.485 |
